# ctDNA transiting into urine is ultrashort and facilitates noninvasive liquid biopsy of HPV^+^ oropharyngeal cancer

**DOI:** 10.1172/jci.insight.177759

**Published:** 2024-03-22

**Authors:** Chandan Bhambhani, Qing Kang, Daniel H. Hovelson, Erin Sandford, Mary Olesnavich, Sarah M. Dermody, Jenny Wolfgang, Kirsten L. Tuck, Collin Brummel, Apurva D. Bhangale, Kuang He, Marc G. Gutierrez, Ryan H. Lindstrom, Chia-Jen Liu, Melissa Tuck, Malathi Kandarpa, Michelle Mierzwa, Keith Casper, Mark E. Prince, John C. Krauss, Moshe Talpaz, N. Lynn Henry, Maria D. Giraldez, Nithya Ramnath, Scott A. Tomlins, Paul L. Swiecicki, J. Chad Brenner, Muneesh Tewari

**Affiliations:** 1 Department of Internal Medicine, Division of Hematology/Oncology,; 2 Michigan Center for Translational Pathology,; 3 Department of Computational Medicine & Bioinformatics,; 4 Department of Otolaryngology,; 5 Department of Internal Medicine,; 6 Department of Pathology,; 7 Department of Radiation Oncology, and; 8 Rogel Cancer Center, University of Michigan, Ann Arbor, Michigan, USA.; 9 Institute of Biomedicine of Seville (IBiS), Hospital Universitario Virgen del Rocío, Consejo Superior de Investigaciones Científicas, University of Seville, Seville, Spain.; 10 Department of Urology,; 11 Department of Pharmacology,; 12 Department of Biomedical Engineering, and; 13 Center for Computational Biology and Bioinformatics, University of Michigan, Ann Arbor, Michigan, USA.

**Keywords:** Oncology, Cancer, Diagnostics, Head and neck cancer

## Abstract

**BACKGROUND:**

Transrenal cell-free tumor DNA (TR-ctDNA), which transits from the bloodstream into urine, has the potential to enable noninvasive cancer detection for a wide variety of nonurologic cancer types.

**Methods:**

Using whole-genome sequencing, we discovered that urine TR-ctDNA fragments across multiple cancer types are predominantly ultrashort (<50 bp) and, therefore, likely to be missed by conventional ctDNA assays. We developed an ultrashort droplet digital PCR assay to detect TR-ctDNA originating from HPV-associated oropharyngeal squamous cell carcinoma (HPV^+^ OPSCC) and confirmed that assaying ultrashort DNA is critical for sensitive cancer detection from urine samples.

**Results:**

TR-ctDNA was concordant with plasma ctDNA for cancer detection in patients with HPV^+^ OPSCC. As proof of concept for using urine TR-ctDNA for posttreatment surveillance, in a small longitudinal case series, TR-ctDNA showed promise for noninvasive detection of recurrence of HPV^+^ OPSCC.

**Conclusion:**

Our data indicate that focusing on ultrashort fragments of TR-ctDNA will be important for realizing the full potential of urine-based cancer diagnostics. This has implications for urine-based detection of a wide variety of cancer types and for facilitating access to care through at-home specimen collections.

**Funding:**

NIH grants R33 CA229023, R21 CA225493; NIH/National Cancer Institute grants U01 CA183848, R01 CA184153, and P30CA046592; American Cancer Society RSG-18-062-01-TBG; American Cancer Society Mission Boost grant MBGI-22-056-01-MBG; and the A. Alfred Taubman Medical Research Institute.

## Abstract 2

A focus on ultra-short DNA is important for robust detection of trans-renal cell-free tumor DNA in urine and should be considered for urine-based cancer diagnostics.

## Introduction

Cell-free tumor DNA (ctDNA) present in biofluids has opened up new opportunities for cancer diagnostics. Although most research in this area has focused on ctDNA present in plasma, ctDNA from the bloodstream can be filtered through the kidney into the urine, as transrenal ctDNA (TR-ctDNA) (1, 2). Whereas urine-based tests for cancer have traditionally been utilized with cancers that directly interface with the urinary tract (e.g., bladder cancer, prostate cancer), TR-ctDNA holds the promise of providing urine-based access to a broad variety of cancer types arising in organs throughout the body (2, 3). Urine offers a completely noninvasive approach that overcomes limits on specimen collection volume and frequency of collection that are associated with blood. Moreover, because urine can be self-collected at home, this remote specimen collection capability could help reach underserved populations and enable more effective cancer screening at a population-wide level.

Although the TR-ctDNA approach holds great potential, there have been mixed reports on the efficiency of TR-ctDNA detection compared with that of blood ctDNA (3–7). A potentially crucial factor for the analysis of TR-ctDNA is knowing the length of TR-ctDNA fragments present in urine, because this affects assay design for optimal sensitivity in TR-ctDNA detection. To date, there have been contrasting reports regarding the length of TR-ctDNA fragments. Whereas PCR-based studies of TR-ctDNA have shown greater success in detection when using amplicons shorter than 60 bp (4, 8, 9), two recent next-generation sequencing (NGS) studies that focused specifically on TR-ctDNA suggested a median length of 112 bp (10) or 101 bp (11), an with additional investigation showing a higher proportion of ultrashort fragments in patients compared with individuals acting as controls (11). A limitation of reported NGS results is that the specific library preparation methods used (e.g., double-stranded DNA [dsDNA] library preparation protocols, hybridization-based capture of ctDNA fragments) are prone to bias against recovery of shorter fragments, especially ultrashort fragments (i.e., <50 bp) (12).

Given that studies of transrenal cell-free DNA (cfDNA) in noncancer settings (e.g., fetal DNA in urine of pregnant woman or *Mycobacterium tuberculosis* DNA in patients with tuberculosis) (13, 14) reported transrenal cfDNA to be ultrashort (<50 bp), we hypothesized that the same may be true for cancer-derived TR-ctDNA in urine. To test this hypothesis, we utilized single-stranded NGS methods capable of capturing the smallest fragments to develop a more complete profile of TR-ctDNA size. As shown in Results, our data indicated that TR-ctDNA is ultrashort (<50 bp) and detectable across multiple nonurologic cancer types.

In addition to the single-stranded DNA (ssDNA) NGS studies, we developed a droplet digital PCR–based (ddPCR-based) assay to measure TR-ctDNA in urine, which offers absolute quantification, higher precision, and higher throughput compared with NGS. We designed this assay to study patients with HPV^+^ oropharyngeal squamous cell carcinoma (OPSCC). In such patients, the HPV DNA sequences are present in the blood circulation as ctDNA, and we hypothesized that such ctDNA fragments that transited the glomerular barrier of the kidney could be detected in urine by ddPCR. HPV ctDNA represents an ideal target for ddPCR assay development for TR-ctDNA, because (a) the sequence of a single HPV subtype, HPV16, is shared by 90% of patients with HPV^+^ OPSCC, and, thus, a single HPV16 TR-ctDNA assay could cover the large majority of patients; (b) since HPV is a nonhuman sequence, the “background” signal from patients without HPV^+^ cancer would be expected to be low; and (c) HPV16 can integrate at multiple sites within the tumor genome, resulting in a higher signal per tumor genome. It is worth noting that unlike the setting of HPV^+^ cervical cancer where shed tumor DNA could be deposited directly into urine, HPV16 signal in the urine of patients with HPV^+^ OPSCC would necessarily be transrenal.

Thus, we sought to develop a first-generation ddPCR assay capable of detecting ultrashort HPV16 TR-ctDNA fragments in urine from patients with HPV^+^ OPSCC. We compared this assay (42 bp amplicon) to a conventional length assay (77 bp amplicon) and found that targeting ultrashort fragments was essential for robust urine TR-ctDNA detection. Utilizing the ultrashort amplicon assay, we achieved TR-ctDNA detection results in urine from patients with HPV^+^ OPSCC that were concordant with results from matched plasma ctDNA. Furthermore, using longitudinal urine samples from a small case series, we showed proof of concept for early detection of cancer recurrence. Thus, our results indicate that by targeting ultrashort DNA fragments, TR-ctDNA becomes a viable approach for HPV^+^ OPSCC detection and potentially for cancer recurrence monitoring after treatment.

## Results

### TR-ctDNA is readily stabilized at the point of collection with EDTA and effectively isolated using a Q Sepharose resin-based strategy.

While some work has been done on urine DNA stabilization and isolation methods (15, 16), there is no accepted consensus yet as to optimal methods, especially with respect to TR-ctDNA. For urine DNA stabilization at the point of collection, we tested the ability of EDTA to effectively preserve spiked-in DNA fragments of varying size in healthy donor human urine. We found that EDTA at a final concentration of 100 mM was able to prevent degradation of all size fragments, compared with the rapid degradation seen within 1 hour when no preservative was added (Supplemental Figure 1, A and B; supplemental material available online with this article; https://doi.org/10.1172/jci.insight.177759DS1). Additionally, we tested the ability of EDTA to stabilize DNA fragments in urine samples for up to 7 days, which is important for being able to use urine samples collected at home and mailed to the lab. We found that the immediate addition of EDTA (final concentration 100 mM) to urine samples at the point of collection preserved DNA fragments of diverse sizes for at least 7 days (Supplemental Figure 1, C and D).

For urine DNA extraction, we tested a previously developed Q Sepharose resin-based TR-ctDNA isolation strategy using an ultralow range DNA ladder spiked into urine samples from people acting as healthy controls (15, 17). We further tested a magnetic bead–based approach to remove high-molecular-weight (HMW) DNA (>500 bp), while retaining lower-molecular-weight DNA (18). We found that these strategies were able to effectively isolate and separate short DNA fragments from urine samples (Supplemental Figure 2).

### TR-ctDNA is ultrashort and detectable in multiple cancer types.

To test our hypothesis that TR-ctDNA is ultrashort, we adopted a ssDNA NGS library preparation protocol that has been used for sequencing of highly degraded “ancient DNA” from fossils. This library preparation method captures ultrashort DNA fragments that are missed by the more commonly used, dsDNA library preparation approaches (12). To determine the size profile of TR-ctDNA in patients with nonurologic cancers, we employed ssDNA library preparation coupled with low-pass whole-genome sequencing (WGS) to characterize cfDNA isolated from 22 urine specimens, representing urine collections from patients with cancer (*n* = 15) with a variety of solid tumors (breast, lung, colon, and rectal) or leukemias (AML). These patients were all known to have ctDNA in the blood, based on detection of tumor-associated copy number alterations (CNA) from sequencing of contemporaneously collected plasma cfDNA.

**Figure 1:**
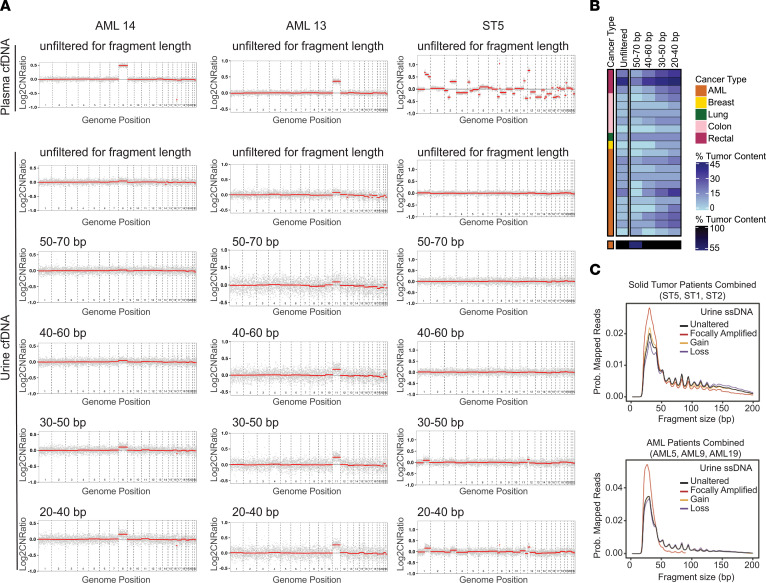
Fragment size analysis of urine cfDNA WGS from patients with nonurologic cancers, showing TR-ctDNA enrichment in ultrashort fragments. (**A**) The plots show log_2_(copy number ratio) (Log2CNRatio) calls on the *y* axis for plasma cfDNA or urine cfDNA for 3 patients, with the *x* axis showing the genomic position of the mapped DNA fragments across the indicated chromosomes. Low-pass coverage urine cfDNA WGS data, unfiltered for fragment length, showing CNA patterns qualitatively concordant with those from matched plasma cfDNA WGS (also unfiltered for length) in some but not all patients; cancer-associated CNAs are visible in patients AML14 and AML13 but not in patient ST5. After stratification of analysis by fragment length into 20 bp wide bins, CNA plots showed that restriction to ultrashort bins (<50 bp; i.e., 30–50 bp and 20–40 bp) revealed tumor-associated CNA more robustly than unfiltered data. (**B**) Heatmap of estimated percentage of tumor DNA content from WGS data. We observed increased enrichment for tumor DNA with ultrashort fragments (i.e., <50 bp) relative to larger fragment size bins, consistent with the qualitative differences evident in **A**. (**C**) Composite curves of urine cfDNA WGS data from 3 patients with solid tumors and 3 patients with AML. The curves labeled as focally amplified, gain, and loss are implicitly derived from tumor-enriched DNA (fragment length of mapped reads emanating from regions with known tumor-associated CNA), while the unaltered curves reflect inferred fragment lengths for reads mapping to the remainder of the genome. As shown, the majority of DNA fragments and, importantly, those mapping to genomic regions of tumor-associated CNA are ultrashort (<50 bp) in length.

We analyzed the urine WGS data, either unfiltered for DNA fragment length or by restricting fragment length to select size bins, by using a peak-calling algorithm to infer genome-wide tumor CNA. The mapping of fragments to regions of CNA served as indication of their tumor-derived origin. Restricting analysis to ultrashort fragments (i.e., 30–50 bp, 20–40 bp length) enhanced the detection of CNA profiles that were concordant with the CNA patterns observed from plasma cfDNA WGS sequencing data (example CNA plots from 3 patients are shown in Figure 1A). Based on the peak-calling algorithm, we were able to also quantify the percentage of DNA fragments corresponding to tumor DNA (based on their mapping to regions of CNA), as has been reported for plasma ctDNA analysis (19). Performing this analysis on the range of fragment size bins showed a progressive enrichment in percentage of tumor DNA in most cases, with ultrashort bins (i.e., <50 bp) showing the highest fractional representation of tumor DNA (Figure 1B).

For 6 of the patients, there were sufficient CNA and read depth to perform a direct fragment length analysis of the genomic regions of cancer-associated CNA, which represent regions that are preferentially derived from tumor DNA in the sample, as compared with nonaltered regions. Through this analysis, we confirmed that the DNA corresponding to cancer-associated CNA regions is ultrashort (<50 bp, with median length ~30 bp) (Figure 1C).

### Ultrashort amplicon ddPCR-based analysis of urine TR-ctDNA detects HPV^+^ OPSCC and shows strong concordance between urine-based and plasma-based ctDNA detection.

Our results suggested that sensitive detection of TR-ctDNA by ddPCR, which offers the benefits of absolute quantification, high precision, and speed, would require assays designed to target ultrashort fragments (i.e., <50 bp). Conventional ddPCR assays for ctDNA target amplicons of more than 50 bp in length, which we hypothesized may miss the majority of TR-ctDNA due to its ultrashort nature. To test this hypothesis, we set out to compare the effectiveness of HPV16 TR-ctDNA detection in urine from patients with HPV16^+^ OPSCC using a custom-designed ultrashort amplicon ddPCR assay versus a conventional length ctDNA ddPCR assay.

**Figure 2:**
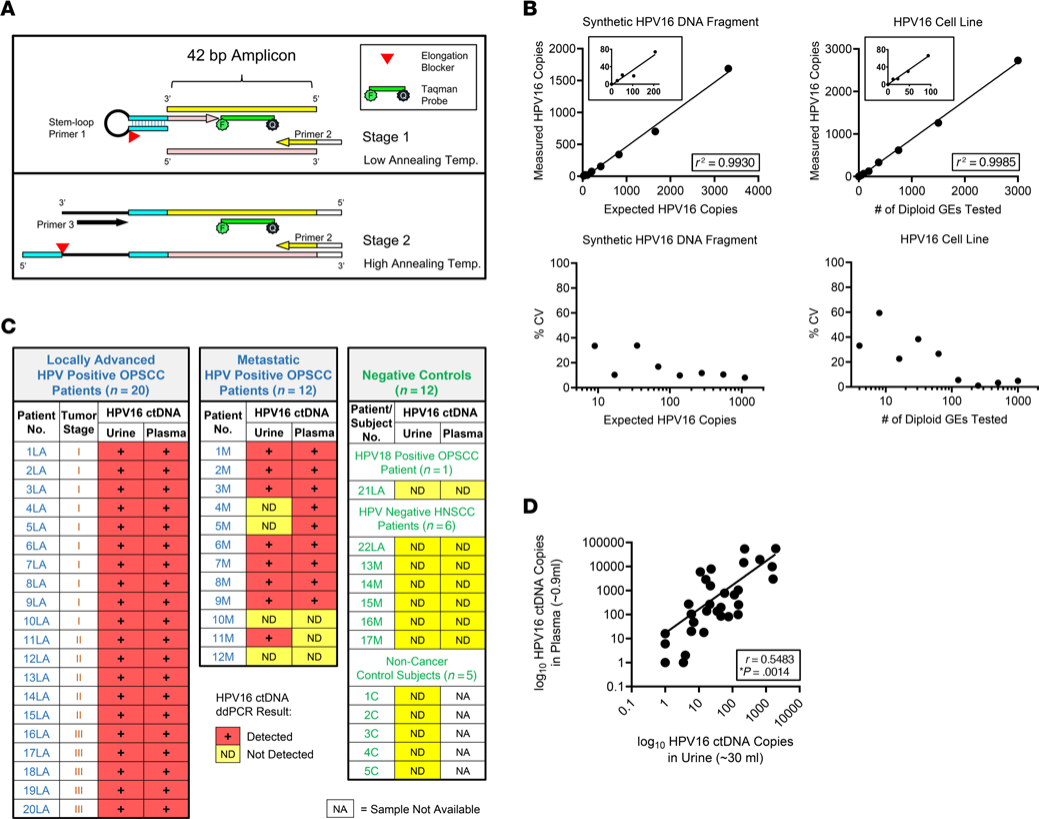
Ultrashort amplicon HPV16 stem-loop ddPCR assay detects TR-ctDNA fragments in urine from patients with HPV^+^ OPSCC and shows concordance with results from plasma ctDNA analysis. (**A**) Schematic of the short-amplicon (42 bp) stem-loop 2-stage PCR approach (17) used to detect and quantify ultrashort HPV16 TR-ctDNA present in urine (see Supplemental Figure 3 for details). (**B**) Analytical validation of the stem-loop assay using a 2-fold dilution series to define the detectable range (top plots). Expected copies of synthetic spiked-in HPV16 target DNA (left) or the GEs of HPV16^+^ cancer cell line UM-SCC-104 tested (right) were plotted against the measured copies (cumulative of triplicates). LoD was determined to be 4.2 copies (see Methods). Bottom plots show observed coefficient of variation (%CV) corresponding to measurement at each dilution of synthetic HPV16 DNA or UM-SCC-104 gDNA. (**C**) Stem-loop assay was used to detect HPV16 ctDNA in matched urine and plasma samples collected before treatment, from 32 HPV^+^ OPSCC patients representing both locally advanced (LA) and metastatic (M) cases, and 12 negative controls (1 HPV18^+^ OPSCC patient, 6 HPV^–^ head and neck squamous cell carcinoma [HNSCC] patients, and 5 healthy individuals without cancer). The assay detected HPV16 ctDNA in both urine and plasma in 27 of the 32 cases of HPV^+^ OPSCC. HPV16 ctDNA was not detected in any of the negative controls (12 of 12 cases), showing high concordance between plasma-based and urine-based ctDNA detection. (**D**) Comparison of log10 HPV16 ctDNA values in urine and matched plasma from 31 patients with HPV^+^ OPSCC (see Methods). Significant correlation was found between HPV16 copies detected in urine and plasma based on Pearson’s correlation test; *P (2-tailed) = 0.0014. HPV16 TR-ctDNA values (cumulative of ddPCR triplicates) correspond to the mean value of 30 mL urine samples from 2 bottles; and ~0.9 ml for plasma samples..

For the ultrashort amplicon assay, we utilized a stem-loop 2-stage PCR approach that allowed for the development of a 42 bp amplicon ddPCR assay (Figure 2A) (17, 20). We designed this to target the HPV16 *E6* gene (Supplemental Figure 3) and measured TR-ctDNA in patients with HPV^+^ OPSCC. The HPV16 *E6* gene represents a highly recurrent ctDNA target in the population of patients with HPV^+^ OPSCC, and HPV16 in general provides a high signal-to-noise ratio because of its low sequence similarity to endogenous human genomic sequences that could be the source of “background” signal.

We performed analytical validation of this assay and found it to have exceedingly low background (i.e., high specificity), providing a limit of detection (LoD) of approximately 4 molecules in a sample and the sensitivity to detect the presence of HPV16 ctDNA at a representation of less than 0.01% of total cfDNA (i.e., 1 copy of HPV16 ctDNA in a background of over 10,000 wild-type human genomes) (Figure 2B). In a series of matched plasma and urine samples (*n* = 32) obtained from patients with either locally advanced or metastatic disease, prior to the start of treatment and known to be p16^+^ in tissue immunohistochemistry (a surrogate for any HPV subtype–associated OPSCC), we found that HPV16 ctDNA could be consistently detected in *both* urine and plasma using the 42 bp amplicon assay in 27 of 32 patients (Figure 2C). In 2 patients, HPV16 ctDNA was detected only in plasma, and in 1 patient it was detected only in urine (Figure 2C). In the remaining 2 patients, HPV16 ctDNA was undetectable in both urine and plasma (Figure 2C). HPV16 ctDNA was undetectable in urine from 11 of 11 individuals acting as controls who did not have HPV^+^ cancer and in 1 control (patient 21LA) with OPSCC related to the HPV18 subtype rather than HPV16 (Figure 2C). For patients with HPV^+^ OPSCC, HPV16 ctDNA abundance values in urine and plasma were found to be positively correlated (Figure 2D).

As an additional confirmation of our conclusion from urine cfDNA-sequencing studies that TR-ctDNA is ultrashort, we compared results from our ultrashort amplicon assay in urine with a validated, conventional length plasma HPV16 ctDNA assay, targeting a 77 bp amplicon (21). As predicted, the ultrashort 42 bp assay detected TR-ctDNA in all patient samples studied, whereas the longer conventional 77 bp assay yielded undetectable or extremely low values of HPV16 TR-ctDNA in all the urine samples (Table 1).

### Proof of concept for urine TR-ctDNA–based posttreatment surveillance for early detection of HPV^+^ OPSCC recurrence.

**Figure 3:**
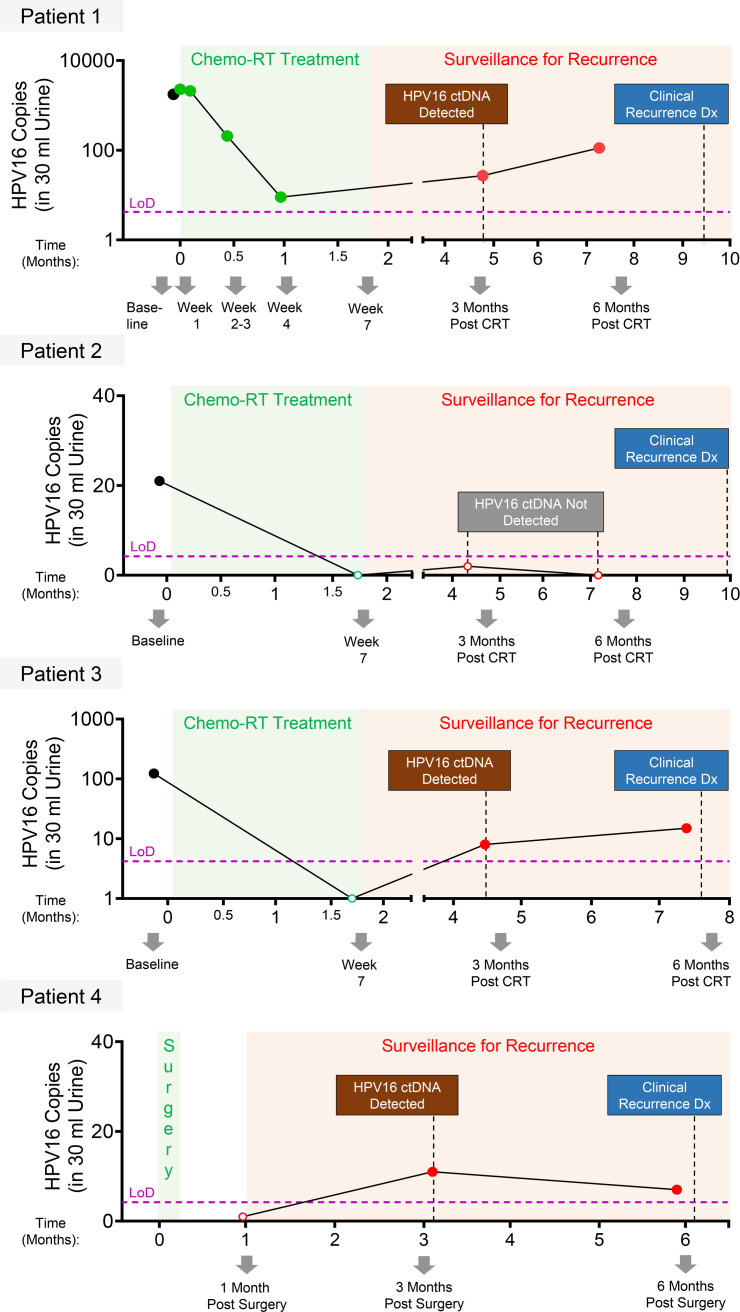
Proof of concept for earlier detection of cancer recurrence via serial urine HPV16 TR-ctDNA measurements in 4 patients with HPV^+^ OPSCC. Shown are ddPCR results from testing of longitudinal urine collections from 4 patients, using the stem-loop 42 bp urine TR-ctDNA assay. Number of HPV16 copies detected (*y* axis) was plotted on a log_10_ scale (patient 1 and patient 3) or linear scale (patient 2 and patient 4) as HPV16 ctDNA values (cumulative of ddPCR triplicates) from urine cfDNA samples collected at different time points over several months (*x* axis). Samples were analyzed from 7 time points for patient 1, 4 time points for patient 2, 4 time points for patient 3, and 3 time points for patient 4. Closed symbols represent HPV16 molecules detected above LoD (magenta dotted line), and open symbols represent values that were below the LoD, with serially collected samples at baseline prior to treatment in black, samples collected during treatment in green, and samples collected during posttreatment surveillance for recurrence in red. The day of treatment is marked as 0 on the *x* axis. Patients 1–3 underwent chemoradiation (CRT) treatment (7 weeks) and showed detectable HPV16 TR-ctDNA prior to initiation of treatment. Patient 4 underwent surgical resection but, notably, had baseline urine DNA of poor quality and, therefore, that time point could not be accurately analyzed for HPV16 TR-ctDNA. In 3 patients, HPV16 TR-ctDNA was detected in urine during the surveillance period prior to clinically detected recurrence.

After clinically validating our HPV16 urine assay in the specimens described above, we assessed the dynamics of urine TR-ctDNA in a small pilot cohort of patients treated for HPV^+^ OPSCC with curative therapy, who had biopsy-proven recurrence within the first year after treatment. To do so, we performed ddPCR on urine samples from 4 patients for whom longitudinal urine samples were available, including at least 1 sample within 3 months prior to the date of clinical recurrence diagnosis by imaging and/or tissue biopsy (Figure 3). Three of these patients had been treated with chemoradiation for locally advanced disease, whereas 1 had been treated by surgical resection. In 3 of the 4 patients (patients 1, 3 and 4), HPV16 TR-ctDNA was detected earlier than clinical diagnosis of recurrence (median of 3.2 months earlier). In the case of patient 2, HPV16 TR-ctDNA was not detected during the surveillance period. However, it is worth noting that urine samples were only available until 2.8 months before clinical recurrence for this patient.

## Discussion

Here, we report that TR-ctDNA is ultrashort and is readily detectable using both NGS and ddPCR approaches when utilizing methods capable of evaluating ultrashort fragments. We demonstrated a ssDNA library preparation and NGS protocol capable of assessing genome-wide somatic copy-number profiles from TR-ctDNA in routine urine samples from patients with hematological and solid tumor malignancies. We determined that the TR-ctDNA signal is enriched when restricting analysis to the ultrashort (20–40 bp) fragments. We then developed and optimized a custom ultrashort stem-loop ddPCR assay that allowed for absolute quantification of tumor-derived ctDNA copies in urine from patients with HPV16^+^ OPSCC with a simpler and faster assay. Furthermore, we showed proof of concept that TR-ctDNA could be used to monitor for and detect cancer recurrence through serial noninvasive urine sampling. This points toward how a urine-based TR-ctDNA assay could be paired with traditional imaging and clinical workflows, opening up the potential to provide earlier detection of recurrence and improved outcomes.

Our work highlights the importance of utilizing detection methods compatible with the ultrashort nature of TR-ctDNA. Compared with dsDNA library preparation approaches that only capture longer DNA fragments, our ssDNA library preparation approach allowed us to capture TR-ctDNA fragments that would otherwise have been missed. Indeed, previous NGS and PCR investigations into TR-ctDNA that reported different results than ours appear to have utilized approaches that did not capture ultrashort fragments (5, 10, 22). We propose that the lack of knowledge about the ultrashort nature of cancer-derived transrenal DNA fragments in urine may be a contributor to the relatively limited study of TR-ctDNA in the literature to date.

Our ddPCR approach demonstrated strong concordance of detection overall between urine and plasma samples for measuring HPV16 ctDNA in patients with OPSCC. Based on our results, we propose a model in which circulating tumor DNA in blood, which at steady-state levels is predominantly in the 150–160 bp size range, is also being processed to smaller fragments (e.g., <50 bp) that are small enough to pass through the glomerular barrier of the kidney into the urine. Our results raise many questions about the nature and mechanism of this process, as well as how it might potentially be enhanced to increase urine TR-ctDNA abundance, for future research. We expect that our urine cfDNA extraction process would avoid detection of HPV DNA from viral particles during active HPV infection, since it removes DNA fragments greater than 500 bp, although future experiments will be needed to determine if this is the case.

Urine has many appealing features as a biospecimen type for cancer detection and monitoring, including that it is a fully noninvasive biofluid that is easy to collect at home and can be shipped to a lab, which could help enable healthcare access to populations of people who face challenges with being able to access healthcare facilities (i.e., phlebotomy). Furthermore, urine can be collected more frequently and at a large volume compared with blood, and generally confers a lower biohazard risk. Frequent collection (e.g., daily) could enable TR-ctDNA kinetics to be used as a high time-resolution biomarker for treatment-response monitoring (23), while collecting larger volumes of urine could be especially important for increasing sensitivity of cancer detection in low-disease-burden applications, such as early detection of cancer, detection of minimal residual disease after therapy, and early detection of disease recurrence (24). Our proof-of-concept demonstration of using a first-generation TR-ctDNA ddPCR assay to detect HPV^+^ OPSCC recurrence supports further studies in this direction.

In future work, it will be important to optimize preanalytic and analytic variables for urine TR-ctDNA analysis, as well as to study biological variation (e.g., day to day and time of day) in urine TR-ctDNA. Furthermore, even our 42 bp stem-loop ddPCR assay is likely missing a large fraction of TR-ctDNA, since our NGS data indicate a median TR-ctDNA fragment size of approximately 30 bp. We noted that there were 2 patient cases in which HPV16 ctDNA was detected in plasma but not in urine, and 1 case where HPV16 ctDNA was detected in urine but not in plasma. In all these cases, the samples had low abundance of HPV16 copies (range of 1–16 copies) and this discordance could be attributed to the high variability of values measured near the LoD (4.2 copies) of the assay. Thus, with improved assays that can detect even shorter fragments (e.g., adapting principles used in approaches for microRNA analysis, where the target nucleic acid is typically ~22 nt long), the sensitivity of the TR-ctDNA approach could be further increased in the future.

In conclusion, we propose that focusing on detecting ultrashort cancer-derived DNA fragments is critical for facilitating the full potential of TR-ctDNA analysis for noninvasive, sensitive, and broadly accessible cancer detection and for monitoring for a variety of clinical applications.

## Methods

### Sex as a biological variable.

Our study enrolled both male and female participants, but sex was not considered as a biological variable.

### Sample collection and cfDNA isolation.

Blood was collected from patients and individuals acting as healthy controls into either K_2_EDTA tubes or Streck Cell-Free DNA BCT. Blood was processed into plasma by double centrifugation at 1,600*g* for 10 minutes, followed by 16,000*g* for 10 minutes, all at room temperature, and stored at –80°C until DNA isolation. Plasma cfDNA was isolated with the QIAamp Circulating Nucleic Acid Kit (Qiagen, 55114).

Urine was collected from patients and individuals acting as healthy controls into 500 mL bottles containing 100 mL 0.5 M EDTA (pH 8.0) as the preservative. Urine supernatant was separated from cellular debris by either double centrifugation at 1,600*g* for 10 minutes, followed by 3,000*g* for 10 minutes, or a single centrifugation at 3,000*g* for 10 minutes, followed by filtering through a 0.45 μm polyethersulfone membrane (e.g., Fisher Scientific, 1690045). The resultant supernatant was either frozen in 50 mL aliquots at –80°C or progressed for urine cfDNA isolation, and frozen at an intermediate step when bound to Q Sepharose resin (Cytiva, 17051001). Urine cfDNA was isolated from a 50 mL aliquot using a Q Sepharose resin-based binding strategy (15, 17, 20) as described below in more detail. A 100 bp synthetic, plant-derived, nonhuman DNA duplex referred to as “plant spike-in,” with a strand sequence of 5′-AGAAGGAGTGACTGATCTTCAACCAGGAGATCATGTTTTACCCATCTTCACCGGAGAGTGTGGGGATTGTCCTCACTGTCACTCCGAGGAATCCAACATG-3′, was spiked into each 50 mL urine aliquot to assess urine cfDNA extraction efficiency. HMW DNA (≥500 bp) was removed using AMPure XP beads, based on a previously reported protocol (18).

### Longitudinal stabilization of cfDNA in urine using EDTA.

Urine samples were collected from participants acting as healthy controls, and 0.5 M EDTA (pH 8.0) was added at the time of collection to a final concentration of 100 mM. A GeneRuler Ultra Low Range DNA ladder was added to the urine sample, homogenized by inversion, and urine was aliquoted into 250 mL tubes stored at room temperature for 0, 3, 5, or 7 days. Day 0 samples had cfDNA isolated immediately. Urine samples were processed, and cfDNA isolated using a resin-based binding strategy described above. DNA ladder bands were measured using a D1000 Bioanalyzer chip.

### WGS.

Low-pass coverage WGS of urine cfDNA was performed using a ssDNA library preparation protocol (25). Corresponding plasma cfDNA low-pass WGS libraries were prepared from matched plasma cfDNA using methods reported previously (19). Data were analyzed using a previously reported pipeline for detecting somatic tumor-associated CNA and estimation of percentage tumor content (also referred to as percentage tumor DNA in the text), which utilized a copy number calling algorithm based on comparison to reference control DNA samples (19).

Low-pass coverage WGS and computational data analysis methods were used to analyze cfDNA isolated from *n* = 15 urine specimens collected from patients with diverse cancer types (multiple samples were collected from some patients, on different days), in whom plasma ctDNA and CNA were known to be detectable by low-pass WGS. Urine WGS results were stratified by inferred cfDNA fragment length into 4 sliding 20 bp wide bins (50–70 bp, 40–60 bp, 30–50 bp, 20–40 bp), and the amplitude of tumor-associated CNA signal in each of them was plotted. For 6 patients for whom there was sufficient read depth and cancer-associated CNA (detected via paired tissue and/or plasma WGS), mapped single-stranded urine cfDNA WGS reads were analyzed by stratifying the inferred cfDNA fragment lengths based on the local CNA status (gain, loss, partially amplified, unaltered).

### Urine cfDNA isolation.

Urine cfDNA was extracted from a 50 mL aliquot using a protocol adapted from a previously described method (17, 20). Briefly, 10 μL of plant spike-in synthetic DNA (at 5,000 copies/μL) serving as urine cfDNA extraction control, and 0.5 mL Q Sepharose resin slurry (Cytiva, 17051001), were added to each 50 mL urine aliquot and rotated at room temperature for 30 minutes. The Q Sepharose resin-bound DNA was collected by centrifugation at 1,800*g* for 5 minutes at room temperature, and supernatant was removed and washed with 4 mL Tris-EDTA (TE) buffer (Fisher Scientific, BP24731). The resin was collected by centrifugation as described above, and excess TE buffer was carefully aspirated out. The resin was then suspended in 0.5 mL of 95% ethanol and frozen at –80°C until DNA isolation.

For DNA isolation, the resin was transferred to a Micro Bio-Spin column (Bio-Rad, 7326204), and the ethanol was removed by centrifugation at 1,000*g* for 30 seconds. The resin was then washed 3 times with 600 μL resin wash buffer (10 mM sodium acetate [pH 5.2] + 0.3 M lithium chloride) by centrifugation for 1 minute at 800*g*. DNA was eluted twice by adding 450 μL resin elution buffer (10 mM sodium acetate [pH 5.2] + 2 M lithium chloride) to the column each time and centrifuging for 3 minutes at 800*g*. Each 450 μL elute was precipitated by adding 3 volumes of 95% ethanol (1.35 mL) and incubating at room temperature for a minimum of 5 minutes. The mixture (700 μL maximum per spin) was applied onto a QIAquick spin column (Qiagen, 28115) for binding of cfDNA by centrifugation at 9,391 g for 1 minute, flow-through was discarded, and the process was repeated until all the mixture was applied. The column was washed twice with 500 μL column wash buffer 1 (10 mM sodium acetate [pH 5.2] + 2 M lithium chloride) in 70% ethanol final) and twice with 500 μL column wash buffer 2 (75 mM potassium acetate [pH 5.0] in 80% ethanol final), followed by centrifugation at 9,391 g for 30 seconds each time. Residual ethanol was removed by centrifugation at 9,391 g for 3 minutes in a fresh collection tube. cfDNA was eluted by transferring the column to a fresh 1.5 mL tube and adding 53 μL buffer EB (Qiagen), followed by centrifugation at 9,391 g for 2 minutes. cfDNA was stored at –20°C or processed for isolation of the low-molecular-weight (LMW) fraction (<500 bp) (18) as described below.

For separation of the HMW DNA fraction (≥500 bp) from the LMW fraction, 50 μL cfDNA elute was incubated with an equal volume of suspension buffer (0.6 M sodium chloride + 16% polyethylene glycol 8000) and 10 μL AMPure XP bead suspension (Beckman Coulter, A63880) for 2 hours at room temperature in a ThermoMixer (Eppendorf), which was programmed to perform agitation at 1,400 rpm for 3 seconds of each 1 minute segment. The AMPure XP beads bound to HMW cfDNA fraction were then pelleted by incubation on a magnetic rack (Thermo Fisher Scientific, 12321D). With the tubes still on the rack, a 105 μL suspension containing LMW cfDNA fraction was carefully transferred to a 1.5 mL tube without disturbing the pellet and kept on ice for further processing. The AMPure XP beads were washed with 150 μL of 75% ethanol and HMW cfDNA fraction eluted in 50 μL TE buffer.

To the 105 μL LMW cfDNA suspension kept on ice, the following were added: 20 μL of 1.5 M sodium chloride, 30 μL of 50 mM magnesium chloride, 1.5 μL of 15 mg/mL GlycoBlue (Thermo Fisher Scientific, AM9516), and finally 300 μL of 100% ethanol; then the suspension was incubated on ice overnight to precipitate the cfDNA. The LMW cfDNA pellet was recovered by centrifugation at 16,100*g* for 30 minutes at 4°C and washed with 80% ethanol by centrifugation at the same speed for 15 minutes at 4°C. The pellet was dried to remove residual ethanol by incubating the tube with the lid open at room temperature for 10 minutes. 25–50 μL buffer EB was added to the pellet (and more specifically for ddPCR, samples were always eluted using 25 μL buffer EB), and it was resuspended by incubation at room temperature. cfDNA was quantified using the Qubit dsDNA HS Assay kit (Thermo Fisher Scientific, Q32854). 19 μL of the 25 μL LMW cfDNA elute was used as template for ddPCR reaction set up in triplicates.

### ddPCR.

All ddPCR experiments were performed using the QX200 Droplet Digital PCR System (Bio-Rad). Each 20 μL reaction mix was partitioned into droplets using the QX200 droplet generator (Bio-Rad), transferred into a 96-well plate, sealed, and cycled in a C1000 Thermal Cycler (Bio-Rad). Droplets were read using QuantaSoft Software in the QX200 reader (Bio-Rad). A short-amplicon 2-stage stem-loop ddPCR reaction was designed as previously described (17) with sequences and PCR cycling conditions provided in Supplemental Figure 3. For the PCR set up, a 21.5 μL sample reaction mix was prepared containing primer 3 and primer 2 at a final concentration of 900 nM each, stem-loop primer 1 and FAM-MGBNFQ probe at a concentration of 250 nM each, and the 1X ddPCR supermix (Bio-Rad, 1863024), of which 20 μL was used for droplet generation. After PCR cycling, the plate was removed from the thermocycler only after the lid temperature fell below 50°C, followed by incubation at room temperature for 5 minutes before proceeding with the droplet reading. The 77 bp HPV ddPCR assay (Table 1) was performed as previously described (21). Primers and synthetic DNA templates were procured from Integrated DNA Technologies, and TaqMan probes were procured from Thermo Fisher Scientific.

### Stem-loop HPV16 TR-ctDNA assay validation and data analysis.

The stem-loop ddPCR assay was validated using a synthetic ultrashort HPV16 DNA duplex with the strand sequence 5′-**AATGC**GTTTCAGGACCCACAGGAGCGACCCAGAAAGTTACCACAGT**TCACT**-3′, which includes 5 bp non-HPV flanking sequences on each end (indicated in bold typeface), or using genomic DNA (gDNA) from the HPV^+^ head and neck cancer cell line UM-SCC-104 digested with HindIII restriction enzyme (21) (Figure 2B). These were tested in a background of HindIII-digested non-HPV human gDNA matrix of 200,000 haploid genome equivalents (GEs) per well. LoD of the stem-loop assay was determined to be 1.4 copies per 20 μL reaction (i.e., 4.2 copies cumulative for ddPCR triplicates) and was calculated using the formula LoD = limit of blank (LoB) + 1.645(SD_low concentration sample_) based on the linearity plots. LoB was determined using 200,000 haploid genomes of HindIII-digested non-HPV human genomic DNA template (blank) background matrix per 20 μL ddPCR reaction set up in triplicates (*n* = 40) using the formula LoB = mean_blank_ + 1.645(SD_blank_).

For urine samples, LMW cfDNA (equivalent to a 30 mL urine aliquot) from 2 different urine collection bottles (for results shown in Figure 2) or from 2–4 bottles per time point (for results shown in Figure 3) was tested in triplicates. Different bottles in this case represent separate urine collections done on the same day or within 3 days of each other for a given time point. Data from each aliquot (representing each bottle) were averaged to determine the HPV16 TR-ctDNA values. There was 1 exception (patient 18LA), for which a sample from only 1 urine bottle was tested (in triplicate ddPCR) (Figure 2, C and D). For plasma samples, cfDNA equivalent to approximately 0.9 mL aliquot was tested in triplicates, and the results were used to compare the HPV16 values in plasma with urine samples (Figure 2, C and D). In 1 case (patient 6M), although both urine and plasma were positive for HPV16 ctDNA, the plasma total cfDNA extracted showed an anomaly: its abundance was 41 SDs higher than the median plasma total cfDNA values from all other patients in the same batch (*n* = 18), and the HPV ctDNA quantification was inaccurate due to signal saturation; therefore, this sample was not included in the correlation analysis (Figure 2D). Of the 5 patient samples with HPV16 ctDNA values below the LoD in urine and/or plasma (Figure 2C), no signal was detected in urine and/or plasma for 4 patients, and a nominal value of 1 was assigned, to be able to plot the correlation on a log_10_ scale (Figure 2D).

### Statistics.

To determine the correlation between HPV16 copies detected in urine TR-ctDNA and plasma ctDNA, statistical analysis was performed using GraphPad Prism 9.0. Two-tailed *P* < 0.05 was considered significant. Pearson’s correlation test was used to analyze statistical values.

### Study approval.

All patients and individuals acting as controls provided written informed consent. This study was approved by the Institutional Review Board at the University of Michigan (protocol nos. HUM00092161 and HUM00066564).

### Data availability.

Values for all data points in graphs are reported in the Supporting Data Values file. Additional data that support the findings of this study will be available upon request, contingent upon review and compliance with government and other pertinent regulations, as well as University of Michigan policies.

## Author contributions

C Bhambhani, QK, DHH, ES equally contributed to the overall primary experimentation reported and drafting of this manuscript. C Bhambhani was listed first because he led the HPV+ OPSCC TR-ctDNA experiments and organized the final manuscript. QK and DHH were listed next, as they initiated this study and led the NGS-based fragment size analysis. QK and ES led the urine cfDNA process development and stability analysis. ES and CB led the patient sample processing and data analysis. C Bhambhani, QK, ES, DHH, KH, NR, M Talpaz, NLH, JCK, SAT, PLS, JCB, and M Tewari contributed to the conception or design of the work. C Bhambhani, QK, ES, MO, SMD, C Brummell, KH, and CJL contributed to data collection. JW, C Brummell, RHL, M Tuck, KLT, SMD, MGG, MK, JCK, M Talpaz, MM, KC, MEP, and NLH coordinated human subjects research, helped curate and/or interpret clinical information, or contributed to patient recruitment for the research. C Bhambhani, QK, DHH, ES, ADB, KH, MDG, NR, SAT, PLS, JCB, and M Tewari analyzed and interpreted the data. QK, C Bhambhani, ES, KH, MDG, and M Tewari drafted the article. C Bhambhani, QK, DHH, ES, KH, RHL, CJL, M Tuck, MGG, MK, MDG, MM, KC, MEP, NR, NLH, SAT, PLS, JCB, and M Tewari critically reviewed the manuscript. All authors approved the final version of the article to be published.

## Supplementary Material

Supplemental data

ICMJE disclosure forms

Supporting data values

## Figures and Tables

**Table 1:**
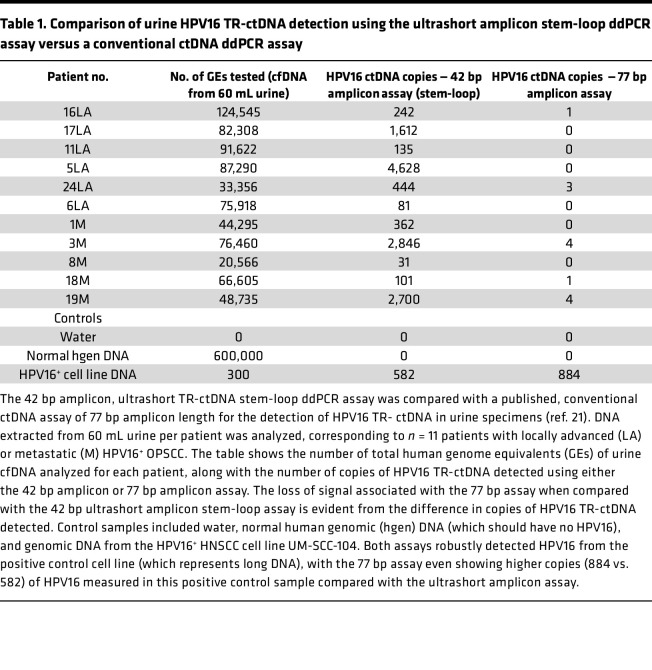
Comparison of urine HPV16 TR-ctDNA detection using the ultrashort amplicon stem-loop ddPCR assay versus a conventional ctDNA ddPCR assay

## Version 1 03/22/2024

Electronic publication
