# Supplementary material for: ctDNA transiting into urine is ultrashort and facilitates noninvasive liquid biopsy of HPV+ oropharyngeal cancer
Source: JCI Insight. 2024 Mar 22;9(6):e177759. doi: 10.1172/jci.insight.177759 (PMC11018327; doi:10.1172/jci.insight.177759)
Supplement: Supplemental data [file jciinsight-9-177759-s251.pdf]

Supplemental Figures

Figure S1

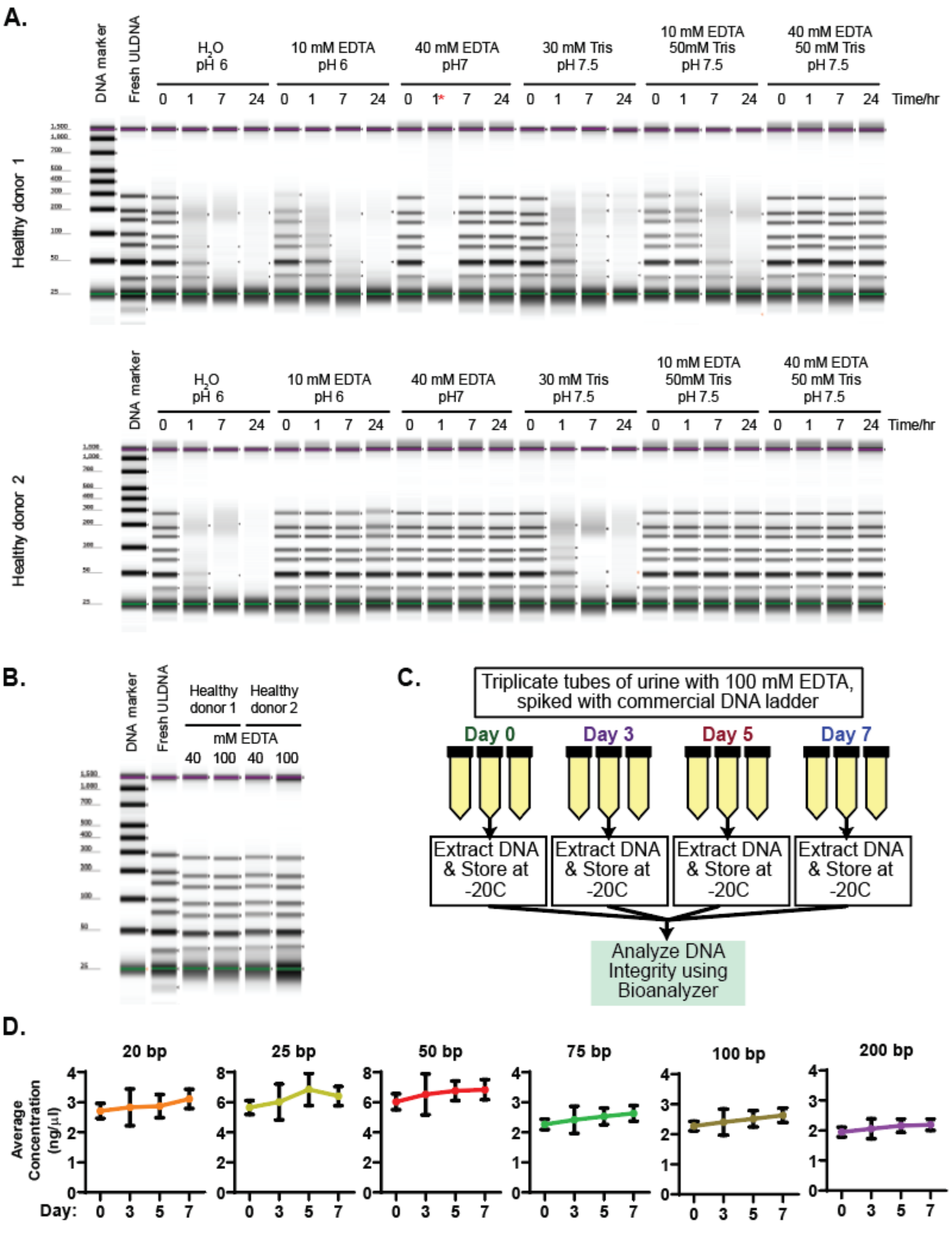

Figure S1. Addition of EDTA to urine stabilizes spiked-in synthetic DNA fragments for at least 7 days when stored at room temperature. (A) GeneRuler Ultra Low Range DNA (ULDNA) ladder (Fisher #SM1212) was spiked into urine samples from two healthy controls, which were then stored at room temperature and preserved with 1) H2O at pH 6.0, 2) 10 mM EDTA at pH 6.0, 3) 40 mM EDTA at pH 7.0, 4) 30 mM Tris-HCl at pH 7.5, 5) 10 mM EDTA/50 mM Tris-HCl at pH 7.5, and 6) 40 mM EDTA/50 mM Tris-HCl at pH 7.5. pH strips were used

to measure the preserved urine. * indicates a lane with error during DNA ladder spike-in extraction. **(B)** Stability of ULDNA ladder was tested after incubation for 24 hours in urine preserved with 40 mM and 100 mM EDTA. Ladder was spiked into the urine collected from two healthy donors in duplicates stored at room temperature and preserved with 40 mM or 100 mM EDTA. **(C)** Schematic of experiment to investigate stability of ULDNA ladder spiked into urine from healthy controls, preserved with 100 mM EDTA and stored at room temperature for up to 7 days. **(D)** Average concentration of individual ULDNA ladder bands as determined by Bioanalyzer for urine left at room temperature for 0, 3, 5, and 7 days.

Figure S2

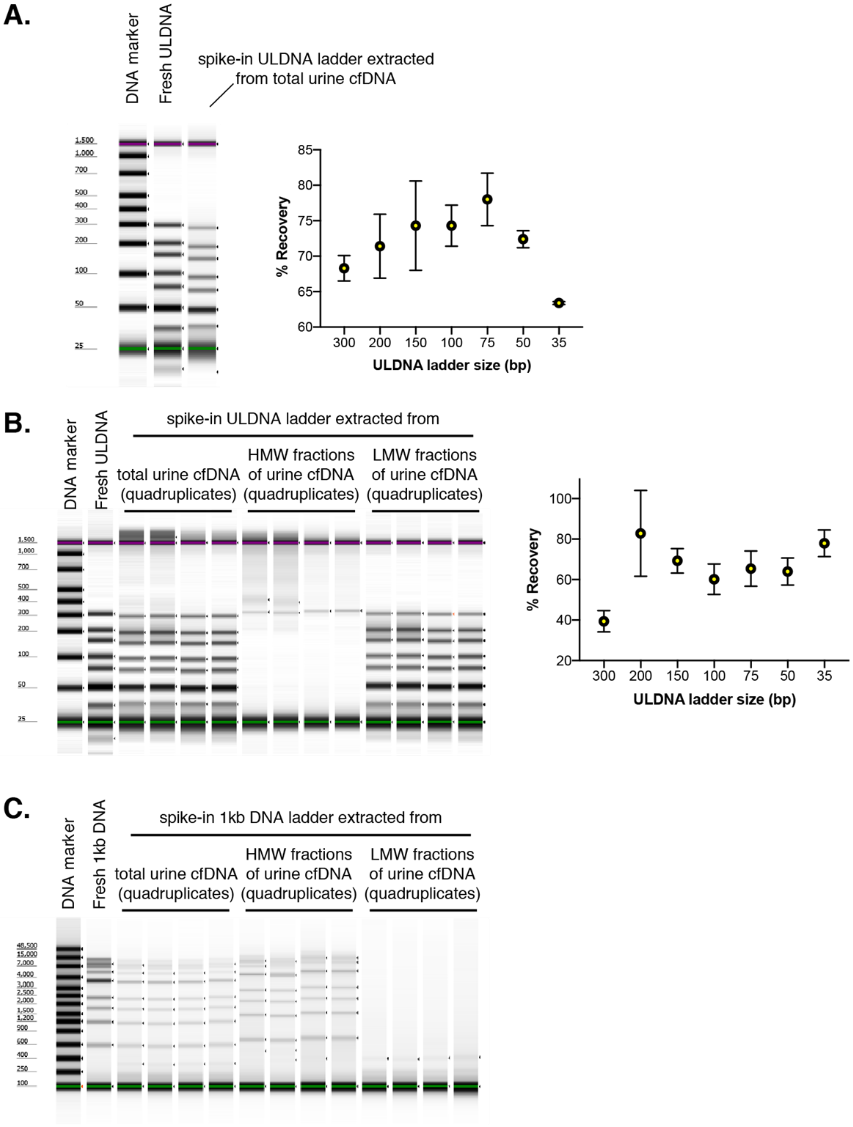

Figure S2. Assessment of efficiency of urine DNA extraction and low MW/high MW DNA separation. (A) Efficiency of DNA extraction of ULDNA ladder from total urine cfDNA. Error bars represent standard deviations of duplicate extractions. **(B)** Efficiency of DNA extraction of Invitrogen 1kb DNA ladder (1kb DNA) from total urine cfDNA, high molecular weight (HMW) fractions of total urine cfDNA, and low molecular weight (LMW) fractions of total urine cfDNA. Error bars represent standard deviations of quadrupole extractions. **(C)** DNA extraction of Invitrogen 1kb DNA ladder (1kb DNA) from total urine cfDNA, HMW fractions of total urine cfDNA, and LMW fractions of total urine cfDNA.

Figure S3

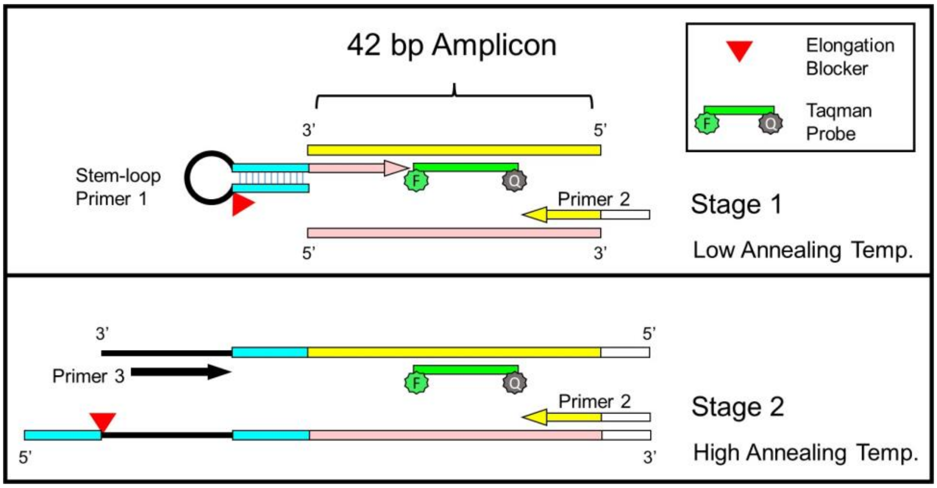

Primer / Probe ID	Sequence (5' to 3')
Stem-loop Primer 1	CCTCAGCCATC/Me-isodC/GTCTGTCCACCGTGAGCTTGATGGCTGAGG AACTGTGGTAACTTT
Primer 2	CGAGACTGTTTCAGGACCC
Primer 3	CTGTCCACCGTGAGCT
Probe	6FAM-CTGGGTCGCTCCTGT-MGB-NFQ
Amplicon +	GTTTCAGGACCCACAGGAGCGACCCAGAAAGTTACCACAGTT
Amplicon -	AACTGTGGTAACTTTCTGGGTCGCTCCTGTGGGTCCTGAAAC

PCR Cycling Conditions for the 2 Stage Stem-loop Assay

Stage	Temperature	Time	Cycles
I	95°C	10 min	1
	94°C	30 sec	10
	49°C	1 min	
II	94°C	30 sec	40
	60°C	1 min	
	98°C	10 min	1
	4°C	Hold	1

All steps were performed with a 2°C/second ramp rate and the lid temperature set to 105°C

Figure S3. Assay design and primer/probe sequences for the HPV16 TR-ctDNA stem-loop ddPCR assay. Stem-loop ddPCR assay design to detect and quantify ultra-short TR-ctDNA fragments present in urine, targeting a short-amplicon (42 bp) region in the HPV16 *E6* gene, with the sequences for the primers, probes and amplicon, and the PCR cycling conditions listed below the schematic. During Stage 1 of the PCR (first 10 cycles), a forward primer (Primer 1; pink) forming a stem-loop (cyan-black) and a tailed reverse primer (Primer 2; yellow), both with short regions of complementarity to the template, anneal at a low

temperature to amplify the ultra-short fragments of the template (+ strand in yellow and - strand in pink). Stage 2 of the PCR (next 40 cycles) unlocks the stem-loop to make a longer version of the template derived from the amplicons in step 1, which can be amplified with higher annealing temperatures using a third reverse primer (Primer 3; black) complementary to the loop region of the stem-loop primer. The elongation blocker 5' modification methyl iso-deoxycytosine (Me-isodC) prevents the amplification of the complementary sequence of the stem (cyan) in Stage 2 resulting in no stem formation at that stage.
